# Species-level determination of closely related araucarian resins using FTIR spectroscopy and its implications for the provenance of New Zealand amber

**DOI:** 10.7717/peerj.1067

**Published:** 2015-07-02

**Authors:** Leyla J. Seyfullah, Eva-Maria Sadowski, Alexander R. Schmidt

**Affiliations:** Department of Geobiology, University of Göttingen, Göttingen, Germany

**Keywords:** Araucariaceae, Amber, New Caledonia, FTIR, New Zealand

## Abstract

Some higher plants, both angiosperms and gymnosperms, can produce resins and some of these resins can polymerize and fossilize to form ambers. Various physical and chemical techniques have been used to identify and profile different plant resins and have then been applied to fossilized resins (ambers), to try to detect their parent plant affinities and understand the process of polymerization, with varying levels of success. Here we focus on resins produced from today’s most resinous conifer family, the Araucariaceae, which are thought to be the parent plants of some of the Southern Hemisphere’s fossil resin deposits. Fourier transform infrared (FTIR) spectra of the resins of closely related Araucariaceae species were examined to test whether they could be distinguished at genus and species level and whether the results could then be used to infer the parent plant of a New Zealand amber. The resin FTIR spectra are distinguishable from each other, and the three Araucaria species sampled produced similar FTIR spectra, to which *Wollemia* resin is most similar. Interspecific variability of the FTIR spectra is greatest in the three *Agathis* species tested. The New Zealand amber sample is similar in key shared features with the resin samples, but it does differ from the extant resin samples in key distinguishing features, nonetheless it is most similar to the resin of *Agathis australis* in this dataset. However on comparison with previously published FTIR spectra of similar aged amber and older (Eocene) resinites both found in coals from New Zealand and fresh *Agathis australis* resin, our amber has some features that imply a relatively immature resin, which was not expected from an amber of the Miocene age.

## Introduction

Resins are secondary metabolites produced in higher plants. Among the gymnosperms the most resinous plants today are found in the conifers, particularly the Pinaceae and the Araucariaceae, although some Cupressaceae can also produce some significant resin amounts (Langenheim, 2003). Tappert et al. (2011) showed that modern conifer resins fall into two broad categories: pinaceous resins and cupressaceous resins, depending on their terpenoid (isoprenoid) composition. Pinaceous resins, from the Pinaceae and *Torreya* (Taxaceae), are based on abietane or pimarane terpenes, whereas cupressaceous resins, which include the Araucariaceae, Cupressaceae, Podocarpaceae and Sciadopityaceae, are based on labdane terpenes. This indicates differences in the terpenoid sythases, which are genetically controlled, and so are of phylogenetic significance (Tappert et al., 2011).

Resins can become fossilized although their potential to do so is directly linked to their terpene chemistry and this varies greatly across conifers. Resin chemistry analyses therefore allow correlations between living plant taxa and can indicate relationships with fossil resinous plants (Lambert, Santiago-Blay & Anderson, 2008).

Resins become fossilized through maturation; this includes their hardening and burial in sediment, where temperature, pressure and permeating fluids affect the rate of chemical transformation (Ragazzi & Schmidt, 2011). Resin maturation is age-related, but it also depends on the thermal history of the resin (Anderson, Winans & Botto, 1992), as well as its chemical structure and composition, since resins are a heterogeneous mixture of chemicals (Langenheim, 2003). Isotopic and chemical changes in amber composition through maturation are minor, except for polymerisation and the loss of volatile components (Nissenbaum & Yaker, 1995; Stout, 1995).

Fossil resins classified as Class I (polylabdanoid diterpenoids), based on the polymeric skeletons of their terpenes (Anderson, 1995; Lambert, Santiago-Blay & Anderson, 2008), comprise the majority of the world’s major amber deposits and thus can be thought to be most chemically similar to the cupressaceous conifer resin type of Tappert et al. (2011). However, the parent plants are still heavily disputed for the largest deposit ever discovered, the Baltic amber succinite deposit, despite being Class 1 (Class 1a: Anderson, 1995) ambers, and various pinaceous, araucarian and sciadopitoid affinities have been suggested (Schubert, 1961; Gough & Mills, 1972; Mosini & Samperi, 1985; Katinas, 1987; Beck, 1993; Langenheim, 1969; Langenheim, 2003; Wolfe et al., 2009) and as yet, none accepted.

Despite the problems of trying to identify the Baltic amber parent plant(s), advances are being made in identifying the parent plant(s) of other major world amber deposits (Penney, 2010). The important amber deposits in the Southern Hemisphere were thought to be mainly araucarian-derived (Lambert, Santiago-Blay & Anderson, 2008) fossil resins (Class 1b: Anderson, 1995). Southern Hemisphere amber has recently been recorded from Peru, South Africa, and Argentina in very small amounts, with more significant amounts found in Australia (Hand et al., 2010) and New Zealand (Kaulfuss et al., 2011).

In Australia amber occurs in Miocene coals (Lyons, Masterlerz & Orem, 2009), and forms the Cape York deposit (post-Jurassic, pre-late Miocene in age: Hand et al., 2010). Amber that has been washed up on southern Australian beaches is not in situ as it has been transported across the ocean (Murray et al., 1994). Various sources for these ambers have been suggested, both an araucarian (Colchester, Webb & Emseis, 2006) and a dipterocarpacean (Sonibare et al., 2014) origin has been postulated. In New Zealand amber is found in Eocene, Oligocene and Miocene sediments and generally, an araucarian origin has been suggested (Thomas, 1969; Lambert et al., 1993; Lyons, Masterlerz & Orem, 2009).

Other Australian ambers tested by Lambert et al. (1993) and Lyons, Masterlerz & Orem (2009), both from southern Australia; and Sonibare et al. (2014, Cape York, northern Australia) appear to have a different botanical source, potentially among the Dipterocarpaceae (Sonibare et al., 2014), although the source area has not yet been identified (Lambert, Santiago-Blay & Anderson, 2008; Lambert et al., 2012; Lyons, Masterlerz & Orem, 2009). Members of the Dipterocarpaceae are some of the most resinous angiosperms (flowering plants) today and their resins can form amber, classified as Class II (polycadinene) fossil resins (Anderson, 1995; Rust et al., 2010).

No Dipterocarpaceae are known in today’s Australian flora, but they are abundant in Southeast Asia, and are thought to have originated in Gondwana in the Late Cretaceous then rafted on the Indian plate and spread out into Asia, based on plant biogeography (Morley, 2000). The dipterocarps have a fossil resin and pollen record stretching back to the Eocene of India (Dutta et al., 2009), however, Australia’s fossil record does not include Dipterocarpaceae, and so the source of the ambers with dipterocarpacean affinities is questionable. Sonibare et al. (2014) suggest transportation of amber from New Guinea despite amber not being known there, or from other Southeast Asian amber deposits. Murray et al. (1994) similarly suggested long distance oceanic transport of dipterocarp resin on to southern Australian beaches. Dipterocarpaceae is not present in the extant flora of New Zealand, nor in its fossil record.

A Podocarpaceae conifer origin for a sole mid-Eocene amber from New Zealand was suggested by Grimalt, Simoneit & Hatcher (1989). This amber was associated with unidentified coalified wood from the Brunner Coal Measures which had the Podocarpaceae pollen *Dacrydiumites mawsonii* (now *Phyllocladidites mawsonii* Cookson 1947 ex Couper 1953) present. Lyons, Masterlerz & Orem (2009) also tested resins from the Eocene bituminous coals of the Brunner Coal Measures of the Reefton Coalfield, but inferred that this amber was more mature *Agathis* amber than younger New Zealand ambers.

Podocarpaceae are not highly resinous today and the family has not been analyzed chemically in detail. Resin only notably occurs in leaves but not from the stems of Podocarpaceae in quantities that would be commercially viable (Langenheim, 2003). There are representatives of Podocarpaceae in both the Australian and New Zealand floras today, and there is a fossil record dating back to the Cretaceous in both Australia and New Zealand (Pole, 1995; Pole, 2000; Parrish et al., 1998).

The majority of New Zealand ambers are thought to have been produced by *Agathis* (Lambert et al., 1993; Lyons, Masterlerz & Orem, 2009). The record of araucarian macrofossils in New Zealand may date back to the Cenomanian (Late Cretaceous, Pole, 2008). *Agathis* fossils are also known from the Eocene fossil record in Australia (Carpenter et al., 2004), and araucarian pollen is known from the Cretaceous of both New Zealand and Australia (Raine, Mildenhall & Kennedy, 2006). However, both Lambert et al. (1993), using NMR C^13^ spectroscopy, and Lyons, Masterlerz & Orem (2009) using FTIR spectroscopy, showed that some Australian ambers tested have a different, but related chemistry to those of New Zealand, potentially indicating a different parent plant species within *Agathis* or the Araucariaceae.

Araucarian conifers today have a Southern Hemisphere distribution and comprise three genera: *Agathis* Salisb., with 21 species, *Araucaria* Juss., with 19 species, and the monotypic *Wollemia nobilis* WG Jones, KD Hill & JM Allen. *Agathis* is the most resinous genus today. There have been some investigations of the resin chemistry of the Araucariaceae (Lyons, Masterlerz & Orem, 2009; Wolfe et al., 2009; Tappert et al., 2011), but to date the sampling within this family has focused on several species of *Araucaria*, the monospecific *Wollemia* and a few *Agathis* species (see Lyons, Masterlerz & Orem, 2009; Tappert et al., 2011).

Several different solid state spectroscopy methods have been used to analyse the bulk chemistry of both resins and ambers, including infrared (IR; e.g., Beck, Wilbur & Meret, 1964), Fourier transform infrared (FTIR; Wolfe et al., 2009), Raman (Edwards, Farwell & Villar, 2007) and ^13^C nuclear magnetic resonance (^13^C NMR; Lambert et al., 1999; Martinez-Richa et al., 2000) spectroscopy. Interestingly, Wolfe et al. (2009) indicated that infra-specific variability in conifer resins was low, meaning that it could be possible to identify different species, perhaps despite quite different local ecological conditions.

Here we use Fourier transform infrared spectroscopy (FTIR) for studying the bulk chemistry of samples of resins across the Araucariaceae following Tappert et al. (2011) to investigate the resin chemistry variability between selected species of Araucariaceae (including some species that were not previously sampled), and to test subsequently whether some Miocene amber from New Zealand, thought to derive from *Agathis*, can be compared to or distinguished from the resins of these extant *Agathis* species that occur close to present day New Zealand.

## Material & Methods

**Figure 1:**
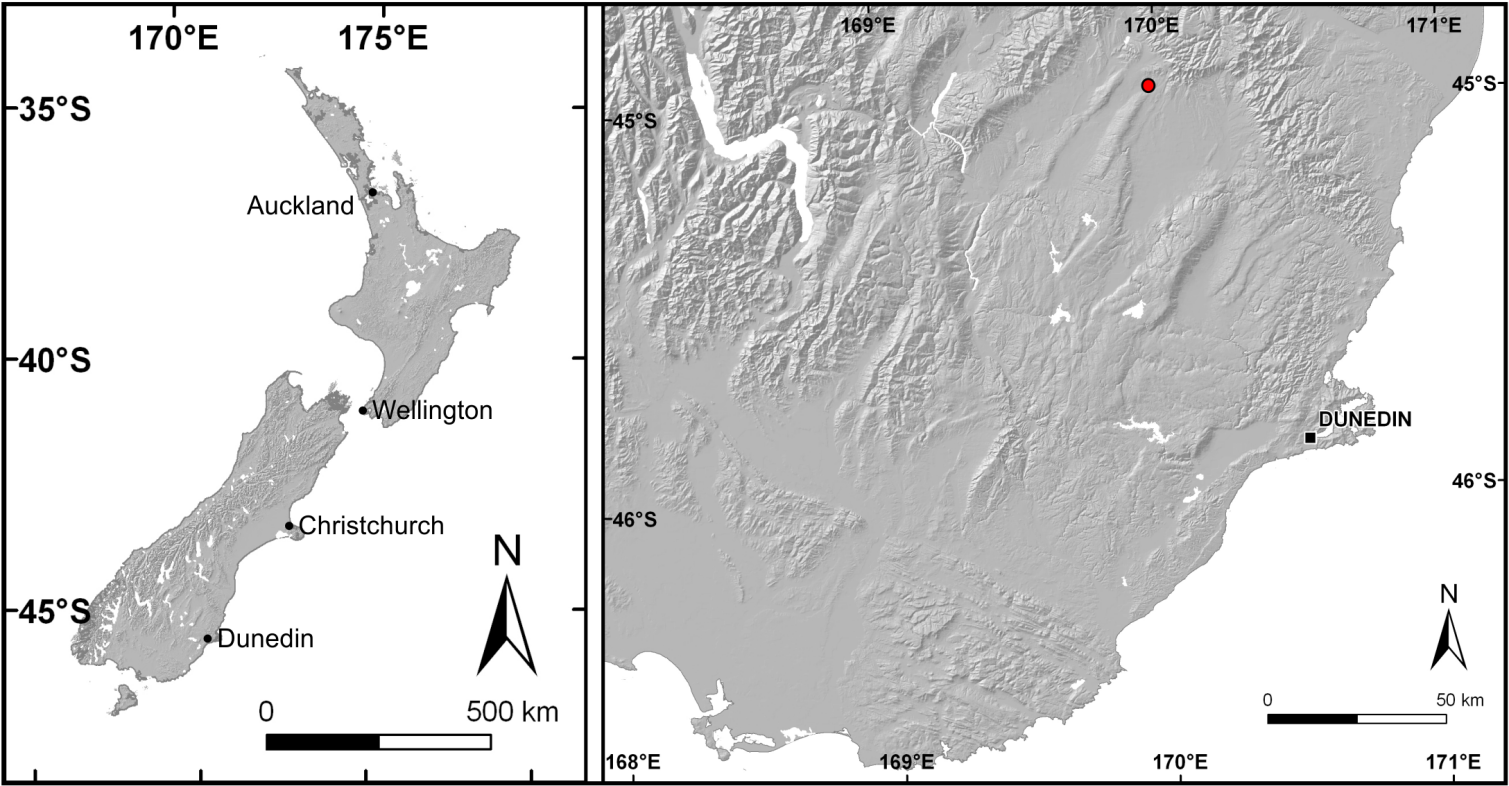
Map of New Zealand and close-up of Otago with Idaburn amber locality (red dot) indicated.

Seven resins from across the Araucariaceae were sampled from wild and cultivated specimens (Table 1). All except the *Araucaria heterophylla* and the *Wollemia nobilis* resins were collected in New Caledonia and New Zealand in 2011, with the two excepted resins collected in 2014. Fieldwork and collection in southern New Caledonia were kindly permitted by the Direction de l’Environnement (Province Sud), permit no. 17778/DENV/SCB delivered in November 2011. Samples were preferentially collected from trunks, if available, but exudates from branches were used if trunk exudates were not available (Table 1). Hardened resin was preferred, but in two cases (*W. nobilis* and *Ar. heterophylla*) semi-solidified resin was collected and prepared. A sample of amber from the early Miocene Idaburn locality in southern New Zealand (Fig. 1) was also tested (see geological information for the amber specimen below). This single large piece of amber was collected in October 2011 and is housed at the Geology Museum of the University of Otago in Dunedin, collection number OU 33159.1. All samples without inclusions or any observable contaminants were chosen, freshly fractured and reduced to a fine powder for application to the central measuring point of the FTIR spectrometer, only tens of micrograms of samples are required per test. Pelletization with KBr was not necessary as the Attenuated Total Reflectance (ATR) technique was used. The absorption spectra were collected in the range 4000–650 cm^−1^ (wavenumbers), equivalent to 2.5–15 µm, using a Jasco 4,100 Fourier transform infrared (FTIR) spectrometer. Spectral resolution was set at 4 cm^−1^. Multiple replicate tests were run per sample until at least three spectra when overlaid were exactly the same, and each time new portions of the ground samples were used. The baseline was not corrected. Bands were identified by comparison with previous reports (e.g., Lyons, Masterlerz & Orem, 2009; Tappert et al., 2011).

**Table 1:** Modern Araucariaceae resins sampled.

Genus, species	Locality collected, date	Location of resin sampled
*Agathis australis* (D. Don) Loud.	Northland New Zealand, 2011	Trunk
*Agathis lanceolata* Warb.	Riviere Bleue, New Caledonia, 2011	Trunk
*Agathis ovata* (C. Moore ex Veill.) Warburg	west of Yaté, New Caledonia, 2011	Trunk
*Araucaria heterophylla* (Salisb.) Franco	cultivated tree, Göttingen, Germany, 2014	Trunk
*Araucaria humboldtensis* J Buchholz	Mt Humboldt, New Caledonia, 2011	Branch tip
*Araucaria nemorosa* de Laub.	Port Boisé, New Caledonia, 2011	Trunk
*Wollemia nobilis* WG Jones, KD Hill & JM Allen	cultivated tree, Göttingen, Germany, 2014	Branch tip

### Geological information for the Idaburn amber sample

**Figure 2:**
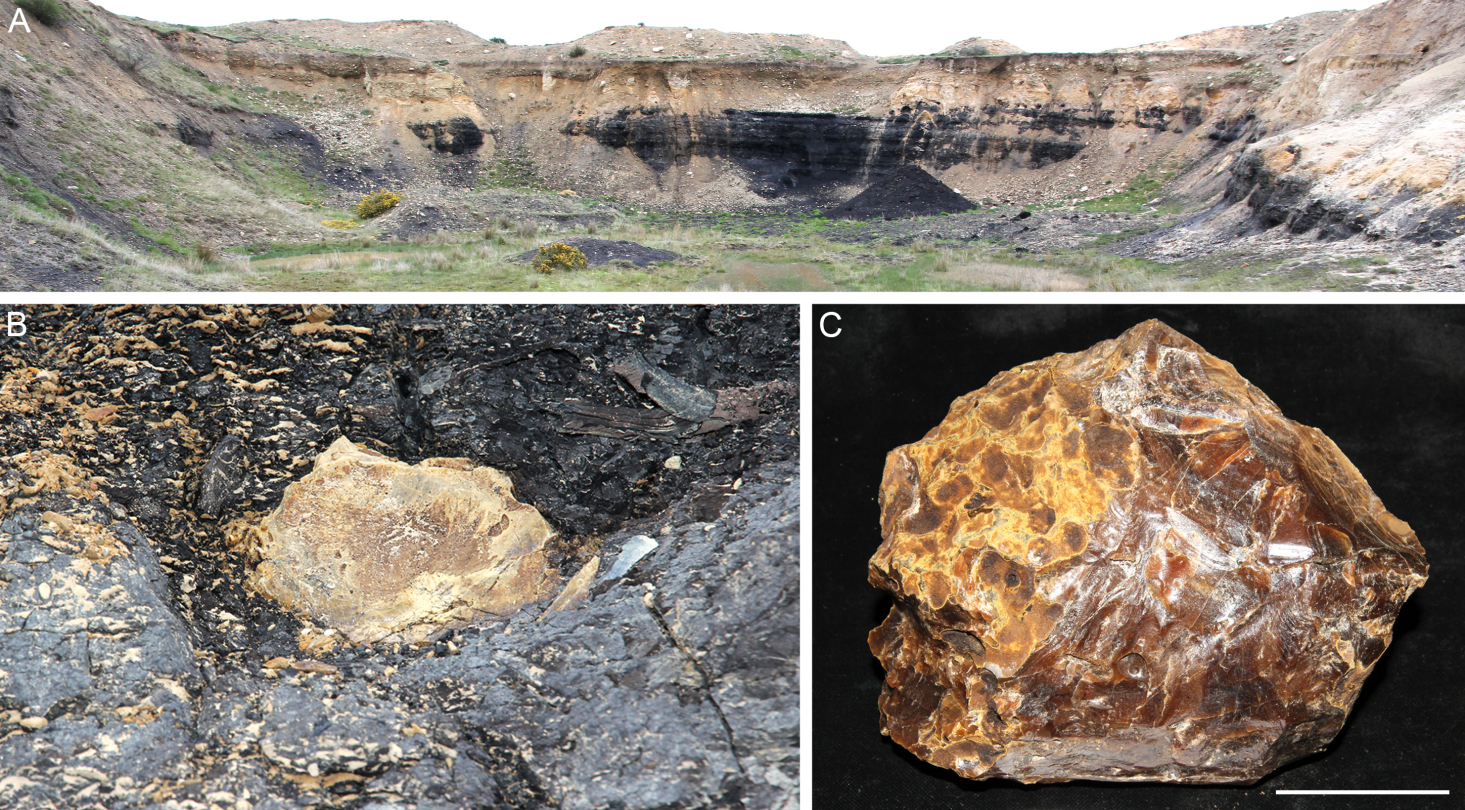
Amber from the former Idaburn Coal Mine, Otago, southern New Zealand. (A) Overview of the exposure of the Oturehua Seam in the Fiddlers Member, Dunstan Formation, from which the amber was collected. (B) In situ amber piece at the exposure of the lignite (Oturehua Seam). (C) Washed amber sample (shown in B) from the same site. Scale is 5 cm.

**Figure 3:**
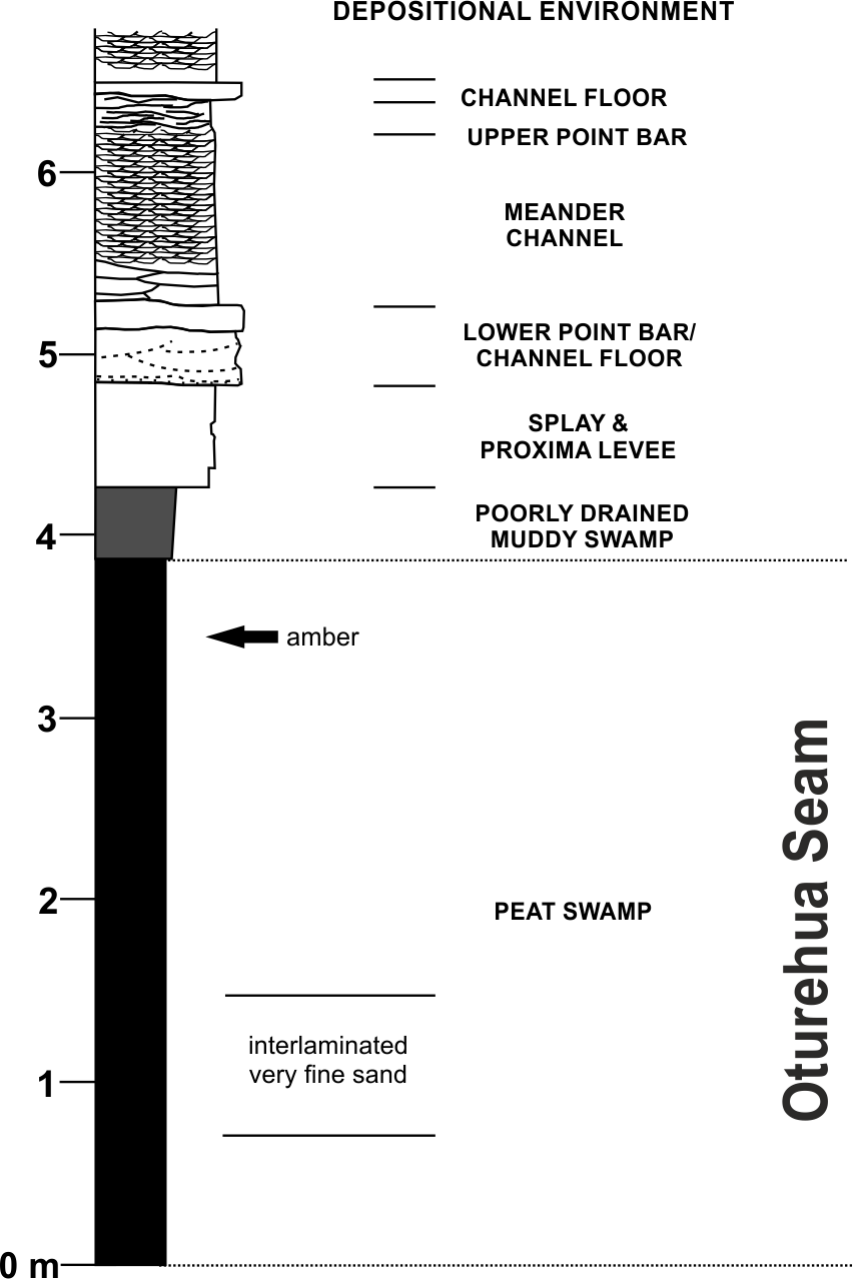
Diagrammatic representation of the exposure at the former Idaburn Coal Mine, Otago, southern New Zealand, showing where the amber was collected, with an interpretation of the depositional environment, redrawn from Lee et al. (2003) with permission.

The amber sample was collected from the disused Idaburn Coal Mine (Fig. 2A), near Oturehua, Central Otago, New Zealand. The GPS coordinates of the site are 44°58′58.63″S 169°58′52.65″E. The sample (Figs. 2B–2C) was taken from the 4 m thick Oturehua Seam (Fig. 3), Fiddlers Member, Dunstan Formation, Manuherikia Group (Douglas, 1986; Lee et al., 2003). The Manuherikia Group consists of fluvial lignite-bearing Dunstan Formation, and the overlying Bannockburn Formation that consists entirely of lacustrine sediments (Douglas, 1986; Lee et al., 2003).

The Fiddlers Member of the Dunstan Formation is widespread in the northern Idaburn district and varies from a few metres to c. 150 m thick. It primarily consists of a fine-grained non-carbonaceous mud-dominated succession with occasional lignite beds. The Fiddlers Member is interpreted as a widespread low gradient flood-basin dominated plain, peripheral to an enlarging lake (Lake Manuherikia), with relatively few river channels (Lee et al., 2003).

The lignite of the Oturehua Seam (Fig. 3) was formed in a swamp forest and includes some beds with relatively high fusain content. There are some horizons with abundant woody remains (including tree trunks and stumps), and beds composed almost entirely of fern-like rachis litter. Very fine sand is found as discontinuous laminae in the lignite (Douglas, 1986; Lee et al., 2003). This means that the amber is considered in situ with no discernable transportation. Interestingly, the lignite has not been very deeply buried, in contrast to other lignites from elsewhere in New Zealand. This is something that could be important in understanding diagenesis (particularly of this amber) and will be investigated further in future research (DE Lee, pers. comm., 2015).

The age of the lignite is early Miocene (Mildenhall, 1989). The amber sample (OU 33159.1, Geology Department collections, University of Otago) used for FTIR analyses was a single in situ piece (Fig. 2B) from near the top of the Oturehua Seam, which was sampled once removed and washed clean (Fig. 2C).

## Results

**Figure 4:**
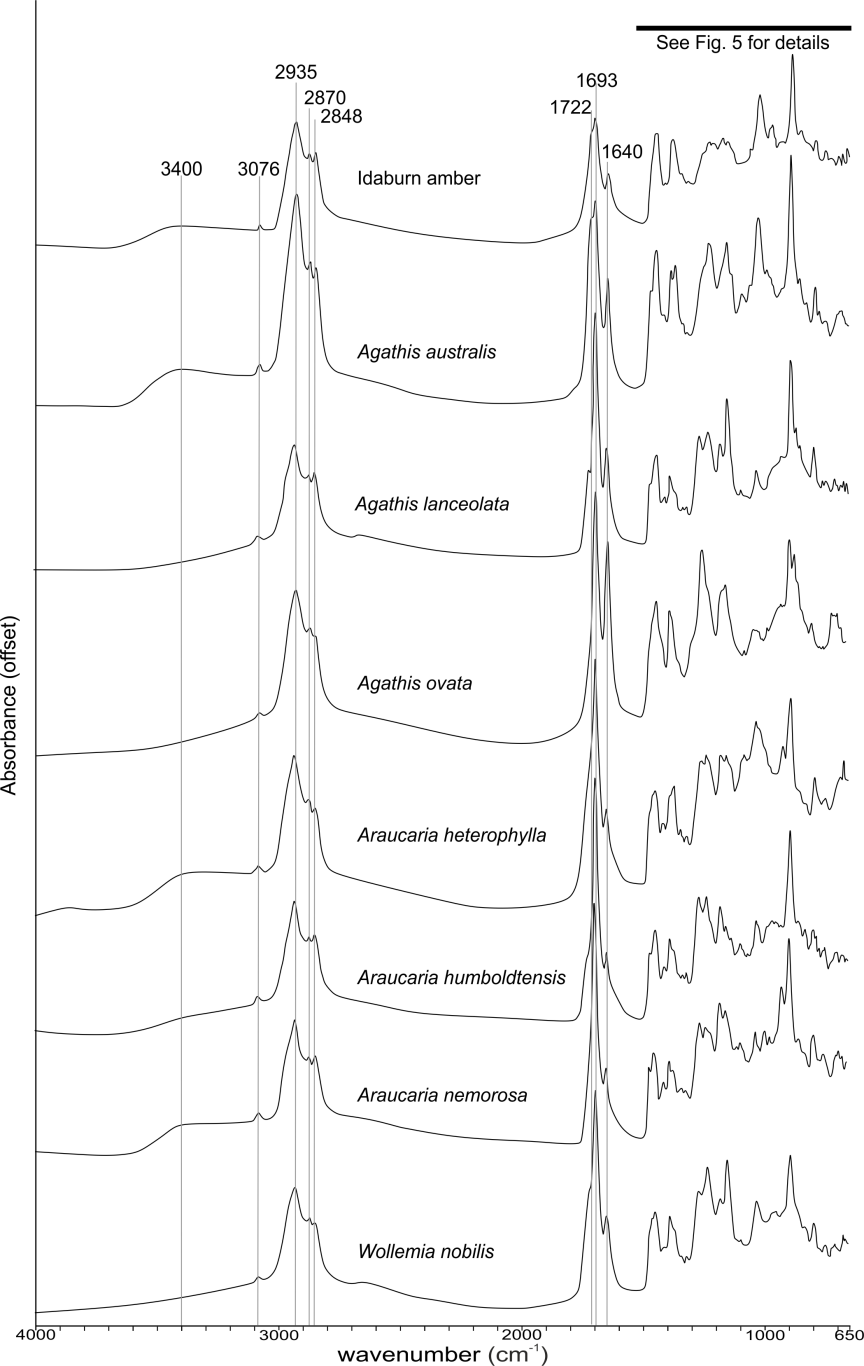
Fourier-Transform infrared (FTIR) spectra of araucarian resins and a Miocene New Zealand amber.

**Figure 5:**
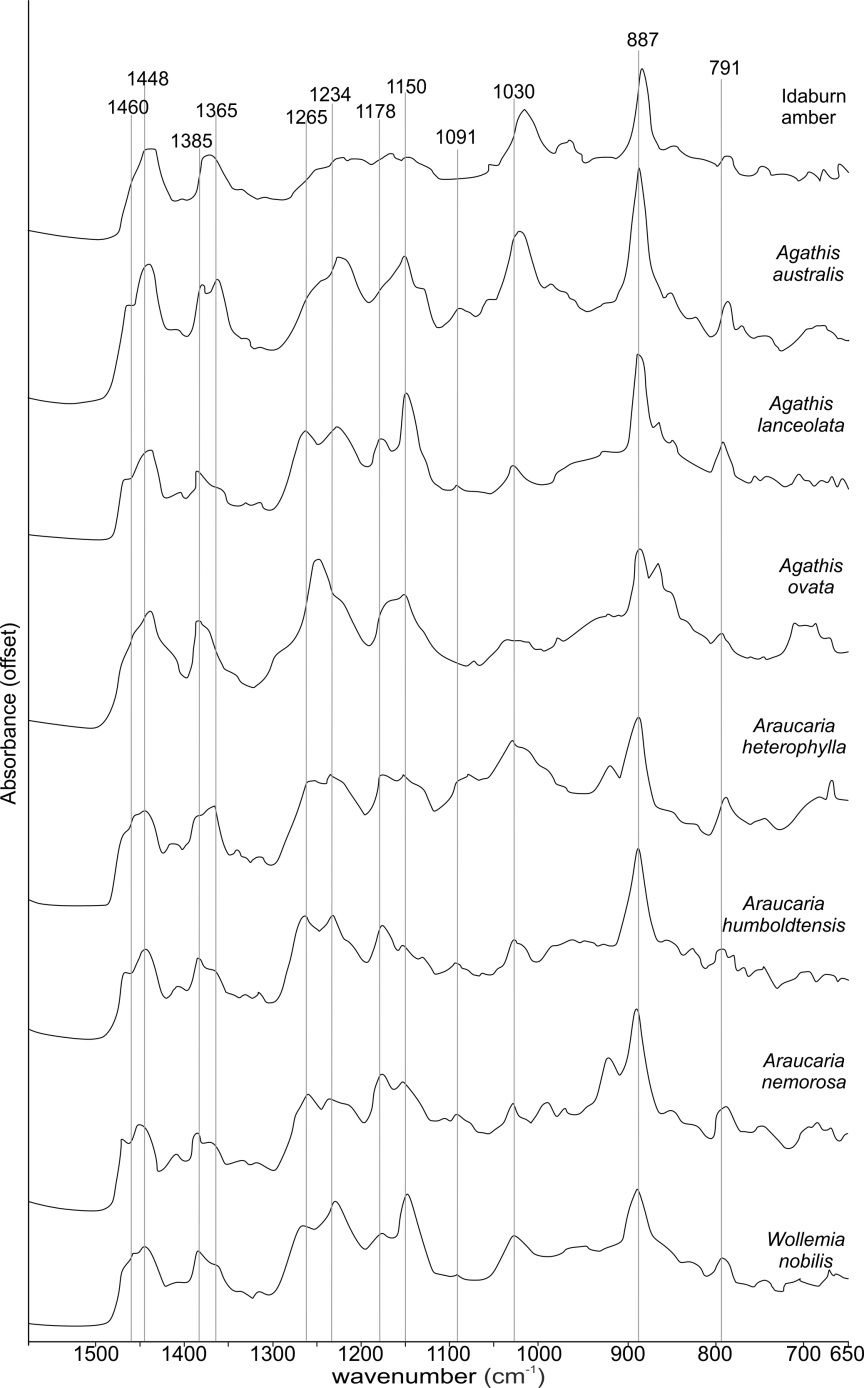
Close-up of the 1,550–650 cm^−1^ spectral region of the Fourier-Transform Infrared (FTIR) spectra of araucarian resins and a Miocene New Zealand amber shown in Fig. 4.

The FTIR results (Fig. 4) show that the seven resins and the Miocene amber from New Zealand are clearly true plant resins, since they appear to have generally similar spectra, but they are distinguishable from each other. Analysis of the key features of the spectra helps to compare and distinguish the resins (Table 2). Moving from left to right across the spectra, key features are highlighted (Figs. 4 and 5). The first is a shoulder generally found around 3,400 cm^−1^ of variable amplitude caused by the stretching of O–H bonds, although it is absent in *Agathis lanceolata, Agathis ovata* and *Wollemia*. All samples share a small peak at 3,076 cm^−1^ caused by the asymmetric C–H stretching of monoalkyl groups and a more prominent peak at around 2,935 cm^−1^ represents a doublet produced by methylene groups, as well as two smaller peaks off the shoulder of the prominent (2,935 cm^−1^) peak at 2,870 cm^−1^ and 2,848 cm^−1^. These three peaks result from aliphatic stretching of single C–H bonds. The 2,870 cm^−1^ peak is associated with methyl groups and the 2,848 cm^−1^ one is a doublet produced by methylene groups.

**Table 2:** Distinctive features of FTIR spectra summarized allowing sample differentiation.

Sample tested	Key distinguishing features (cm^−1^)											
	3,400	1,722	1,460	1,385	1,365	1,265	1,234	1,178	1,150	1,091	1,030	791
Idaburn amber	s	s	-	s	s	-	-	-	-	-	off	s
*Agathis australis*	s	s	T	p	p	-	s	s	p	p	off	s
*Agathis lanceolata*	-	s	T	p	s	p	p	p	p	p	p	p
*Agathis ovata*	-	-	-	p	s	-	-	s	p	-	wide	p
*Araucaria heterophylla*	s	-	-	p	p	p	p	p	p	s	p	p
*Araucaria humboldtensis*	s	s	T	p	s	p	p	p	p	p	p	p
*Araucaria nemorosa*	s	-	T	p	s	p	p	p	p	p	p	p
*Wollemia nobilis*	-	s	-	p	s	p	p	p	p	p	p	p

**Notes.**

p peak s shoulder - no feature present T trough wide relatively wider peak off offset peak from measurement

The next peak shared by all taxa is at 1,693 cm^−1^, with a weak shoulder present at around 1,722 cm^−1^ for some taxa (amber sample, *Agathis australis*, *Wollemia*, *Araucaria humboldtensis* and *Agathis lanceolata*), both are related to the C = O bonds in the carboxyl groups of resin acids. A smaller peak shared by all taxa at 1,640 cm^−1^ is probably related to O–H bending bond (Tappert et al., 2011) or to exomethylene (Lyons, Masterlerz & Orem, 2009). The next section (between 1,550–650 cm^−1^), known as the fingerprint region is shown in detail (Fig. 5), as there are many peaks and troughs here. At 1,460 cm^−1^ *Agathis australis*, *Agathis lanceolata*, *Araucaria humboldtensis* and *Araucaria nemorosa* have a small trough on the shoulder of the peak at 1,448 cm^−1^, which is shared by all samples. These features are related to C–H bending motions of methyl and methylene functional groups.

The 1,385 cm^−1^ peak is shared by all samples, except for the amber sample where this is a shoulder to a peak at around 1,375 cm^−1^, *Agathis australis* which also has a second equal peak at around 1,365 cm^−1^, and *Araucaria heterophylla*, which also has a slightly stronger second peak at around 1,365 cm^−1^, the other resins have a small shoulder at around 1,365 cm^−1^.

The peaks between 1,300–1,100 cm^−1^ are features assignable to C-O single bonds, with the next peak occurring at 1,265 cm^−1^, except for *Agathis australis*, *Agathis ovata* and the amber sample. All samples except that of the amber, *Agathis australis* and *Agathis ovata* share a peak at 1,234 cm^−1^, and all samples except *Agathis australis*, *Agathis ovata* and the amber, have a peak at 1,178 cm^−1^, whereas both *Agathis australis* and *Agathis ovata* have a shoulder and the amber may be interpreted to have a shoulder to a very small peak. All samples, except for the amber share a peak at 1,150 cm^−1^. All samples then have a tiny peak at 1,091 cm^−1^, except the amber and a shoulder instead for *Araucaria heterophylla*. The next, larger peak is at 1,030 cm^−1^ present in all samples except those of *Agathis ovata*, where it is a wider, shallower peak, and amber, where the peak appears offset, occurring at around 1,012 cm^−1^.

The next peak shared by all samples is at 887 cm^−1^ which is attributed to the out-of-plane C–H bending motions in terminal methylene groups. Both *Araucaria heterophylla* and *Araucaria nemorosa* have a smaller peak preceding this at around 930 cm^−1^, on the shoulder of the peak at 887 cm^−1^. The final feature of interest is a peak at 791 cm^−1^, shared by all samples’ spectra except those spectra for the amber and *Agathis australis* samples, which have a shoulder rising to a peak at around 780 cm^−1^ instead.

The overall picture in terms of spectra from the resins of extant araucarian trees is that the three *Araucaria* species are the most similar to each other as they each have nine distinguishing features in common. *Wollemia* and the three *Araucaria* species share eight distinguishing features, and the greatest variability in distinguishing features is seen within the three *Agathis* species (Table 2).

The Idaburn amber sample is similar in key (shared) features with the resin samples (Figs. 4 and 5), but does differ from the extant resin samples in some of the distinguishing features (Table 2), particularly at 1,385 cm^−1^, with a shoulder instead of a peak, and having no distinctive peaks between 1,265–1,091 cm^−1^, unlike the resin spectra, which is most likely due to the different chemistry that has resulted from fossilization/polymerization. In terms of comparing the amber to the resins tested here, the amber shared most features with that of *Agathis australis* (Figs. 4, 5 and Table 2).

## Discussion

Comparing our results with that of Tappert et al. (2011) shows that our spectrum for *Agathis australis* is highly comparable to theirs, although there are some minor intensity differences. Our *Wollemia* spectrum is broadly similar, except that we do not have the shoulder at around 3,400 cm^−1^. This is most likely due to a difference in age or freshness between our sample and that of Tappert et al. (2011), and therefore the degree of polymerization, as suggested by Tappert et al. (2011). Mustoe (1985) states that while these hydroxyl groups may be structural components of the resin, they may also be from water vapour absorbed from the atmosphere. KBr pelletization saturates samples and can affect this signal, but the variation we see is unlikely to be due to sample preparation since both we and Tappert et al. (2011) did not need to use KBr pelletization. Diagenetic alteration can be excluded from our modern resin samples.

Wolfe et al. (2009) stated that the intensity of the C = O absorbance at 1,600–1,800 cm^−1^ and that of C–H at 1,300–1,500 cm^−1^ are related and modulated by the samples’ oxidation history, but they also show that these spectral regions are still potentially useful in discriminating modern resin samples, where little oxidation would have occurred. Thus this potential alteration in the C = O absorbance at 1,600–1,800 cm^−1^ and of C–H at 1,300–1,500 cm^−1^ spectral regions must be taken in to account when comparing FTIR spectra of modern resins directly with fossilized ones. Polymerization reduces the number of hydroxyl (OH) groups, as well as having effects on other groups, and this can be seen to affect the intensity of the 3,400 cm^−1^ region. The 3,400 cm^−1^ region may also be affected by diagenetic alteration and by use of KBr pelletization (see below for the discussion about the Idaburn amber).

The differences could also possibly be due to differences in the environment surrounding the trees when they produced the resins, which could potentially affect the primary resin chemistry. The similarities of our spectra with those of Tappert et al. (2011) lead us to conclude that the spectra for our samples are comparable and expand our knowledge on araucarian resin FTIR spectra.

The relatively low variability of the *Araucaria* species’ resins could be due to the low sample size across this genus of 19 species. Previous work by Tappert et al. (2011) sampled *Araucaria laubenfelsii*, and the published spectrum indicates potentially more variability across this genus. The greater variability of FTIR spectra within *Agathis* species shown here may also indicate that further sampling of araucarian species is needed to map the full FTIR spectral variation of both *Araucaria* and *Agathis* species. Additional samples from other parts of the plants should also be included to understand any potential variation in an individual of a species. Intraspecific variation of these species has not yet been tested, but Wolfe et al. (2009) showed that FTIR spectra of *Pinus* has greater interspecies variation than intraspecific variation across Canada.

Cupressaceous resins, which derive from Araucariaceae, Podocarpaceae, Sciadopityaceae and Cupressaceae, are mainly diterpenoid and have similar FTIR spectra, Tappert et al. (2011) state that they are not distinguishable, based on a very small sample number illustrated. This study has however shown that some bulk chemistry differences within the resins of the Araucariaceae are detectable and that there is much more variation between species than expected. This means that FTIR can be used as a first step to assess the similarity/differences of closely related resins and can be helpful in making an assessment of their interspecific variation.

A second application would be the detection of the first changes denoting polymerization of resins (Tappert et al., 2011). This means that resin FTIR spectra could be used to guide more intensive and expensive subsequent physical and chemical identification work (e.g., ^13^C NMR, Pyrolysis gas chromatography mass spectrometry). Here we show that the New Zealand amber sample is quite distinct in our dataset, and our results support an *Agathis* affinity as there was most similarity to *Agathis australis* resin, which has been previously suspected of being the parent plant of New Zealand ambers (Lambert et al., 1993); but the sample size is too small to confirm this and a potentially extinct parent plant of the Araucariaceae cannot yet be ruled out. Pollen records show that *Araucariacites australis* Cookson has been present in Australia and New Zealand since the Cretaceous (Raine, Mildenhall & Kennedy, 2006), but this pollen could represent extinct species of both *Agathis* and *Araucaria*. Macrofossil evidence supports the presence of both *Agathis* and *Araucaria* in southern New Zealand in the late Oligocene to early Miocene (Lee, Bannister & Lindqvist, 2007; Jordan et al., 2011). The third Araucariaceae genus, *Wollemia* may also have been present in New Zealand from the Jurassic to the early Miocene, based on distinctive pollen, although this pollen type could also have been produced by other *Agathis* species (Jordan et al., 2011; MacPhail & Carpenter, 2013).

Lyons, Masterlerz & Orem (2009) compared modern *Agathis australis* resin with various Southern Hemisphere resinites (amber fragments inside coals) of Eocene to Miocene age. Our amber spectrum has overall similarities to all of theirs, but ours lacks clear peaks between about 1,265–1,091 cm^−1^, and we suspect that this reflects an effect of maturation, a diagenetic alteration of the fossil resin.

Our spectrum of modern *Agathis australis* is more similar to the New Zealand resinite samples than to those Australian ones of Lyons, Masterlerz & Orem (2009). They considered the Australian resinites to have a different botanical origin from the *Agathis* source of the New Zealand resinites. Interestingly our amber spectrum has some further features that imply a relatively immature amber. Fossil resins undergo structural and compositional changes as a consequence of the effects of increasing degrees of maturation (e.g., Anderson, Winans & Botto, 1992). In Class 1b fossil resins, to which the Idaburn amber is thought to belong, this is most apparent by the loss of exomethylene through isomerization (Beck, Wilbur & Meret, 1964; Beck et al., 1965; Beck, 1986; Anderson, Winans & Botto, 1992; Anderson, 1995; Clifford & Hatcher, 1995).

The presence of exomethylene groups are peaks at around 3,082 cm^−1^, 1,644 cm^−1^ and 887 cm^−1^ (Anderson, Winans & Botto, 1992; Lyons, Masterlerz & Orem, 2009), seen in all samples tested here, although slightly less intense in the amber sample. Lyons, Masterlerz & Orem (2009) showed that exomethylene (C = CH_2_) amounts in New Zealand fossil resinites decrease with maturation, with the exomethylene peaks becoming less distinct, eventually disappearing in more mature material. The Idaburn amber sample shows fairly strong exomethylene signals (particularly when compared to the Miocene amber FTIR spectra of Lyons, Masterlerz & Orem, 2009), indicating a relatively immature fossil resin, which is unexpected since the in situ Idaburn amber is Miocene in age (Mildenhall, 1989).

Other effects of maturation may be seen in FTIR spectra of the Idaburn amber sample when compared with the resin samples. Wolfe et al. (2009) stated that the absorbance of C–H at 1,300–1,500 cm^−1^ are related and modulated by the samples’ oxidation history, possibly explaining why there is a shoulder at 1,385 cm^−1^ rather than the peaks seen in all the resin samples. Absorbance peaks between 1,265 cm^−1^ and 1,091 cm^−1^ indicate C–O bonds, and these are subdued or absent in the amber, but various peaks are seen here in the resins.

It may be that the relatively shallow burial of the lignites of the Dunstan Formation (DE Lee, pers. comm.) is being at least partially reflected in the apparent low maturity of the Idaburn fossil resin, when compared to ambers from other localities in New Zealand where the lignites are known to have been more deeply buried.

## Conclusions

This is the first study to apply FTIR spectroscopy to resins produced across closely related members of the Araucariaceae from *Agathis* and *Araucaria* plants growing in New Zealand and New Caledonia, and *Wollemia* from Australia (but grown in Germany). FTIR spectra of resins sampled across the Araucariaceae show unexpected variation, despite the small sample size: environmental variation could be a reason for the variability, but the spectra also show that the species’ resins are similar in chemical composition. When the resin FTIR spectra are compared with a Miocene New Zealand amber sample, a clear relationship is supported showing that the amber is indeed a fossilized Araucariaceae plant resin, but a contradiction appears since the amber has some features of a (relatively) immature fossil resin, particularly when compared to other fossil resins of the same age from New Zealand, perhaps indicating differences in diagenetic histories.

Further investigation is needed to better understand the chemistry of New Zealand amber. FTIR is a very simple, cheap and efficient method for detecting bulk chemistry of both resins and ambers, and needs very little preparation and sample size, making it an excellent first step in the physical and chemical analyses of resins.

## Supplemental Information

10.7717/peerj.1067/supp-1 Supplemental Information 1 Collection permit for New Caledonia

## Additional Information and Declarations

### Field Study Permissions

The following information was supplied relating to field study approvals (i.e., approving body and any reference numbers): Fieldwork and collection in southern New Caledonia were kindly permitted by the Direction de l’Environnement (Province Sud), permit no 17778/DENV/SCB delivered in November 2011.
