# Supplementary material for: Species-level determination of closely related araucarian resins using FTIR spectroscopy and its implications for the provenance of New Zealand amber
Source: PeerJ. 2015 Jul 2;3:e1067. doi: 10.7717/peerj.1067 (PMC4493646; doi:10.7717/peerj.1067)
Supplement: Supplemental Information 1 [file peerj-03-1067-s001.pdf]

PROVINCE SUD
NOUVELLE-CALÉDONIE

RÉPUBLIQUE FRANÇAISE

PRÉSIDENCE

SECRÉTARIAT GÉNÉRAL

AMPLIATIONS

Commissaire délégué	1
DENV	1
Intéressé	1

N° 3287-2011/ARR/DENV

du : 7 NOV. 2011

ARRÊTÉ

accordant à Monsieur Vincent PERRICHOT une autorisation relative à l'accès et à l'utilisation de ressources biologiques, génétiques et biochimiques

LE PREMIER VICE-PRÉSIDENT DE L'ASSEMBLÉE DE LA PROVINCE SUD

Vu la loi organique modifiée n° 99-209 du 19 mars 1999 relative à la Nouvelle-Calédonie,

Vu le code de l'environnement ;

Vu le contrat accessoire passé entre la province Sud et le récolteur ;

Vu le rapport n°2055-2011/ARR du 4 novembre 2011,

ARRÊTE

ARTICLE 1 : Monsieur Vincent Perrichot est autorisé à récolter les échantillons listés ci-dessous et à les utiliser à fins de recherche scientifique, et les transporter, sous réserve de l'observation des prescriptions énoncées à l'article 3.

Famille	Genre	Espèce	Site de récolte envisagé (avec indication du propriétaire foncier)	Type d'échantillon souhaité	Quantité nécessaire (nombre ou poids)
Araucariaceae	*Araucaria*	*columnaris*	Port Boisé	cônes mâles et femelle, feuilles, coulées de résine	1à2/ 5à10/ 50
Araucariaceae	*Araucaria*	*humboldtensis*	Mt Humboldt		
Araucariaceae	*Agathis*	*ovata*	Col de Yaté, plaine des lacs, Madeleine, lac en 8, PPRB, Dzumac, Koghis		
Araucariaceae	*Agathis*	*lanceolata*			
Araucariaceae	*Agathis*	*moorei*			
Cycadaceae	*Cycas*	*spp.*	Port Boisé		
Podocarpaceae	*Dacrydium*	*araucarioides*	Col de Yaté, plaine des lacs, Madeleine, lac en 8, PPRB, Dzumac, Koghis		
Podocarpaceae	*Dacrydium*	*balansae*			
Podocarpaceae	*Dacrydium*	*lycopodioides*			
Podocarpaceae	*Falcatifolium*	*taxoides*			
Podocarpaceae	*Dacrycarpus*	*vieillardii*			
Podocarpaceae	*Acmopyle*	*pancheri*			
Champignons résinicoles	*Indéterminés*	*Indéterminés*	Mt Humboldt, Port Boisé, Col de Yaté, plaine des lacs, Madeleine, lac en 8, PPRB, Dzumac, Koghis	Fragments de quelques cm	2 à 3 échantillons
Cryptogames					
Arthropodes	*Indéterminés*	*Indéterminés*	Mt Humboldt, Port Boisé, Col de Yaté, plaine des lacs, Madeleine, lac en 8, PPRB, Dzumac, Koghis	Quelques spécimens	Quelques spécimens

__ARTICLE 2__ **:** L'autorisation d'accès aux ressources est valable pour les sites mentionnés au tableau ci-dessus et jusqu'à la date du 31 décembre 2011.

Les aires protégées suivantes sont concernées : Mont Humboldt, chutes de la Madeleine et parc provincial de la Rivière Bleue.
Les espèces protégées suivantes sont concernées : *Cycas spp.*
Remarque : cette espèce est inscrite au tableau II de la convention CITES

__ARTICLE 3__ **:** Le projet décrit dans la demande susvisée doit être réalisé conformément aux conditions suivantes :

- La collecte au sein des réserves naturelles ou parcs n'est autorisée que pour les espèces dont les seuls peuplements connus ne se trouvent qu'au sein d'une ou plusieurs aires protégées.
- Sur un même lieu de collecte, et afin de maintenir les possibilités de régénération naturelle, l'intéressé s'engage à ne pas prélever, pour une même espèce, plus de 20% des graines disponibles (cônes femelles).
- Déposer tout holotype afférent à la mission au Muséum d'Histoire Naturelle à Paris.
- Dans le cadre de l'amélioration des connaissances et du suivi des espèces rares et menacées, le rapport annuel des collectes devra mentionner les coordonnées GPS des lieux de collecte, à minima pour les espèces protégées par le code de l'environnement de la province Sud (citées à l'article 2) ainsi que l'espèce suivante : *Agathis moorei.*

__ARTICLE 4__ **:** Une exonération des frais de dossier est accordée.

__ARTICLE 5__ **:** Le présent arrêté sera transmis à Monsieur le commissaire délégué de la République et notifié à l'intéressé.

Pour le président et par délégation
Le directeur de l'environnement

J. FOURMY

PROVINCE SUD

REPUBLIQUE FRANCAISE

DIRECTION DE L'ENVIRONNEMENT

19, avenue Foch, immeuble Foch, 2e étage
BP 3718 - 98846 Nouméa cedex – Nouvelle-Calédonie –
Tél : 24 32 55 – email : denv.contact@province-sud.nc
Fax : (687) 24 32 56

CONTRAT AUTORISANT
L'ACCES A DES RESSOURCES BIOLOGIQUES, GENETIQUES ET BIOCHIMIQUES ET EN FIXANT LES CONTREPARTIES
N° 17778 .

Entre :

La province Sud, représentée par son président, M. Pierre FROGIER,
agissant ès qualité au nom et pour le compte de l'assemblée de la province Sud,
ci-après dénommé "Le propriétaire foncier"

d'une part,

et :

........GEOSCIENCES RENNES - CNRS - UNIVERSITE RENNES 1........,
siège social : ....
~~ridet~~ ....Siret n° 193 509 361 00161...............,
représentée par ....VINCENT PERRICHOT...
dûment habilité aux fins des présentes,
ci-après dénommé "Le récolteur"

d'autre part,

Préambule

Le code de l'environnement de la province Sud met en œuvre le principe de partage juste et équitable des bénéfices découlant de l'utilisation des ressources génétiques établi par la Convention sur la Diversité Biologique et les lignes directrices de la Convention de Bonn.
Désormais, le Titre 1er du Livre III, et plus précisément les articles 312-1 et 312-4 réglementent la collecte de ressources biologiques, génétiques et biochimiques. Lorsqu'une une autorisation provinciale préalable est requise, elle est conditionnée par la signature d'un contrat définissant les conditions d'accès à la ressource.

Article 1 – Objet

Le présent contrat a pour objet de définir les modalités d'accès et de récolte de ressources génétiques, biochimiques ou biologiques sur un terrain appartenant au propriétaire foncier et de préciser les compensations financières et non financières concédées en contrepartie de l'accès accordé aux ressources.

Article 2 – Récolte des ressources

Le propriétaire foncier autorise le récolteur à procéder à la collecte des espèces listées en annexe 1, dans les conditions fixées en annexe 2.

Le récolteur s'engage à informer le propriétaire foncier des dates et horaires des opérations de récolte dès qu'ils sont précisément établis.

Le récolteur s'engage à recourir aux méthodes de collecte garantissant le moindre impact sur la faune et la flore en présence.

Article 3 – Obligations des parties

Le récolteur s'engage à ce que soit fait mention de l'origine des échantillons dans toute publication découlant directement des collectes effectuées dans le cadre du présent contrat.

Le propriétaire foncier s'engage à ne pas utiliser ou divulguer toute information figurant aux annexes 1 et 2.

Article 4 – Propriété des résultats – brevet

Le présent contrat n'ouvre aucun droit de propriété sur les droits découlant des résultats des travaux scientifiques conduits par le récolteur, au-delà des compensations financières et non financières décrites à l'article 5.

Article 5- Compensations financières et non financières de l'accès aux ressources

Le récolteur tiendra, au 31 mars de chaque année, un décompte annuel des revenus générés durant l'année fiscale précédente par ses travaux rendus possibles par la récolte effectuée dans le cadre du présent contrat.
Le récolteur concède, conformément aux dispositions des articles 313-3 du code de l'environnement de la province Sud une contrepartie financière égale à 2 % du montant des ventes des produits dérivés de la ressource collectée avant imposition.
Les versements s'effectuent au 31 mai de chaque année sur la base du décompte annuel.

Article 6 – Responsabilités

Le récolteur devra indemniser le propriétaire foncier de tous préjudices, directs ou indirects, résultant de son fait.

Article 7 – Condition suspensive et résolutoire

Le présent contrat ne prendra effet qu'à la date de délivrance de l'autorisation au récolteur pour l'accès aux ressources biologiques, génétiques et biochimiques concernées par le présent contrat.

Article 8 – Modification du contrat

Le présent contrat pourra être modifié par avenant, à la demande de l'une ou l'autre des parties, dans le respect des dispositions du titre 1er du Livre III du code de l'environnement susmentionnées.

Article 9 – Résiliation du contrat

En cas de non-respect par l'une des parties, des engagements respectifs inscrits dans le présent contrat, celui-ci pourra être résilié de plein droit par l'une ou l'autre des parties à l'expiration d'un délai d'un mois suivant l'envoi d'une lettre recommandée avec accusé de réception valant mise en demeure d'avoir à remplir ses obligations

Toutefois, le présent contrat pourra être résilié de plein droit et sans délai, en cas de retrait de l'autorisation provinciale.

Article 10
Le présent contrat est établi en deux exemplaires originaux.

Article 11 – Lois applicables
Le présent contrat est soumis aux lois et règlements applicables en Nouvelle-Calédonie.

Article 12 – Compétence juridictionnelle
Les litiges éventuels entre les parties relatifs à l'existence, la validité, l'interprétation, l'exécution et la résiliation du présent contrat (ou de l'une quelconque de ses clauses), que les parties ne pourraient pas résoudre à l'amiable, seront portés devant la juridiction compétente de Nouvelle-Calédonie.

Fait à ....Rennes................,

le ....24/09/2011..........

En deux exemplaires originaux.

**Le propriétaire foncier**	**Le récolteur**
Pour le Président et par délégation<br>Le Directeur de l'Environnement<br>J. FOURMY	

Annexe 1 Plantes

Famille	Genre	Espèce	Type d'échantillon souhaité	Quantité nécessaire (nombre ou poids)
Araucariaceae	- Araucaria<br><br>- Agathis	- columnaris<br>- humboldtensis<br><br>- ovata<br>- lanceolata<br>- moorei	feuilles + 1-2 cônes mâles et femelles + résine ⇓ pour chaque espèce	5-10 feuilles 1-2 cônes 10-20 coulées ⇓ pour chaque espèce
Podocarpaceae	- Dacrydium<br><br>- Falcatifolium	- araucarioides<br>- balansae<br>- lycopodioides<br>- taxoides	idem	idem
	- Dacrycarpus<br>- Acmopyle	- viellardii<br>- pancheri	idem	idem

Annexe 2 Plantes

Espèces	Date ou période de récolte envisagée	Nom des personnes chargées de la récolte
	du 5 au 23 Novembre 2011	- Vincent Perrichot (Univ. Rennes 1 - France)
		- Alexander Schmidt (Univ. Göttingen Allemagne)
		- Leyla Seyfullah (Univ- Göttingen Allemagne)
		- Christina Beimforde (Univ- Göttingen Allemagne)

(Insectes) +

Annexe 1 (Cryptogames et champignons)

Famille	Genre	Espèce	Type d'échantillon souhaité	Quantité nécessaire (nombre ou poids)
- Insectes vivant à proximité des arbres résinifères	- indéterminés -	indéterminées	Insectes capturés puis conservés dans l'alcool	1-2 insectes de chaque espèce présente
- Champignons résinicoles + Cryptogames	- indéterminés -	- indeterminées -	fragments de quelques cm -	- 2-3 échantillons de chaque espèce présente sur les arbres résinifères

Annexe 2

Espèces	Date ou période de récolte envisagée	Nom des personnes chargées de la récolte
- insectes -	du 5 au 23 Novembre 2011	- Vincent PERRICHOT
- Cryptogames + champignons	du 5 au 17 Novembre 2011	- Jouko RIKKINEN (Finlande)
